# Robotic vs. open ureteral reimplantation: A retrospective comparative single‐centre series

**DOI:** 10.1002/bco2.70110

**Published:** 2025-11-16

**Authors:** Alice Bourillon, Lucas Freton, Gregory Verhoest, Juliette Hascoet, Claire Richard, Camille Haudebert, Romain Mathieu, Lee C. Zhao, Karim Bensalah, Benoit Peyronnet

**Affiliations:** ^1^ Centre Hospitalier Universitaire de Rennes Service d'Urologie Rennes France; ^2^ NYU Langone Health New York New York USA

**Keywords:** reimplantation, robotics, ureter, ureteroneocystostomyopen

## Abstract

**Objectives:**

To compare the outcomes of open versus robotic ureteral reimplantation procedures at a single centre.

**Material and methods:**

The charts of all patients with ureteral strictures who underwent open ureteral reimplantation between 2005 and 2024, and those who underwent robotic reimplantation between 2013 and 2024, were retrospectively reviewed, and the outcomes of the two approaches were compared.

**Results:**

Eighty patients were included in the final analysis: 45 in the open surgery group and 35 in the robotic group. After a median follow‐up duration of 16,5 months for the open group and 10 months for the robotic group, the stricture recurrence rate was similar in both groups (6,8% vs. 8.6%; p = 0.99). In terms of long‐term complications, there were similar rates of symptomatic reflux (4.4% vs. 5.7%; p = 0.99) and flank pain (8.9% vs. 8.6%; p = 0.99) between both groups. There were more recurrent urinary tract infections in the open group (17.8% vs. 8.6%) and more de novo lower urinary tract symptoms in the robotic group (11.4% vs. 2.2%), but these differences were not statistically significant (p = 0.33 and p = 0.16, respectively). Overall, 30 patients (38%) had at least one long‐term complication (35.6% vs 40%; p = 0.82). The only variable significantly associated with the risk of stricture recurrence was radiotherapy (OR = 11.2; p = 0.01).

**Conclusion:**

The robotic approach appears to be non‐inferior to the open approach in terms of stricture recurrence while being associated with a shorter length of hospital stay and lower estimated blood loss. More importantly, the present series raises questions regarding the long‐term consequences of ureteral reimplantation and confirms the higher risk of failure in radiated patients.

## 1 INTRODUCTION

Ureteral stricture is a common condition that every urologist is likely to encounter during his/her career. Although the exact prevalence is difficult to determine, reported incidences are approximately 0.3–2.5% after gynaecologic surgery, 3% following ureteroscopy and about 1.2% after pelvic radiotherapy.^1^, ^2^

Ureteral reimplantation is the standard procedure for treating distal ureteral stricture.^3^ Like many reconstructive procedures, it was initially performed via open surgery, and evolved with surgical advancements—laparoscopic reimplantation was first reported in 1994, followed by the first robotic‐assisted procedure in 2003.^4^ Despite these developments, as of 2022, the majority of ureteral reimplantations in the United States were still conducted using the open approach.^5^ Currently, no official guidelines recommend specifically the robotic approach, and the open approach remains the gold standard.

Numerous studies comparing laparoscopic and robotic ureteral reimplantation—particularly for reflux treatment in paediatric populations—are available in the literature.^6^ Conversely, only a handful of studies have aimed to compare the outcomes of open versus robotic ureteral reimplantation for ureteral strictures in adults.^7^, ^8^ The findings of those series are often controversial, particularly regarding operative time and the actual advantages of robotic surgery. Differences in hospital stay duration, blood loss and complication severity remained inconclusive.

Therefore, the objective of this study was to compare the outcomes of open versus robot‐assisted ureteral reimplantation for ureteral stricture in adults.

## 2 MATERIAL AND METHODS

### 2.1 Study design

All adult patients who underwent ureteral reimplantation for distal ureteral stricture at a single academic centre between 2005 and 2024 were considered for inclusion.

Patients treated for vesicoureteral reflux were excluded, as this condition was regarded by the authors as a distinct pathology involving a different patient population and clinical presentation. Surgical approaches other than open and robot‐assisted were also excluded.

The patients were retrospectively identified using the Classification Commune des Actes Médicaux (CCAM) codes JCEA001, JCEA002, JCEA004, JCEA005 or JCMA004 and were screened for eligibility in the institution's database. The following techniques were included: ureteral reimplantation without bladder mobilization (ureteroneocystostomy), either refluxing or non‐refluxing; side‐to‐side reimplantation; and psoas hitch reimplantation, either refluxing or non‐refluxing. Procedures not involving ureteral reimplantation—such as ureteroenteric reimplantation—were excluded. Boari flaps were also excluded. The following baseline characteristics were collected for each patient: age, gender, body mass index (BMI), past medical history, prior radiotherapy, previous treatments for the stricture, characteristics of the stricture (length, location), preoperative assessment, duration of ureteral rest (defined as the number of days with no double J stent prior to the surgical repair), use of indocyanine green, intraoperative ureteroscopy.

This study was conducted in accordance with the Declaration of Helsinki on ethical principles for medical research. **This study was approved by the** CNIL (Comité National Informatique et Liberté) **under the reference number 2235966.**

### 2.2 Surgical techniques

#### 2.2.1 Robotic reimplantation

**The patient was placed in a 20° to 30° Trendelenburg position with spread legs. Four robotic ports were placed according to the standard set‐up for robotic pelvic surgery. One 12 mm assistant port was used, and an extra 5 mm assistant port was added on a case‐by‐case basis. The DaVinci Si, X and Xi were used in all cases in the present series with a transperitoneal approach.**

While intraoperative ureteroscopy and intravenous indocyanine green were never used at the beginning of the inclusion period, these tools were used routinely in recent years. **Intraoperative ureteroscopy was used to guide the ureteral dissection with the ureteroscope introduced either through the nephrostomy tract in an antegrade fashion or through the urethra in a retrograde fashion. The stricture was located thanks to the ureteroscopy and IV indocyanine green was given in several cases to check the blood supply and excise the poorly vascularized tissue.** The ureter was always meticulously dissected free from surrounding tissues, minimizing coagulation to preserve its vascular supply.

The use of an antireflux technique was documented whenever performed. When an antireflux mechanism was created, it was always a Lich‐Gregoir with the opening of the detrusor on 4 cm and the opening of the bladder mucosa only on the distal 1 cm of the detrusorotomy. The ureter was spatulated, and its distal end was anastomosed to the bladder mucosa. The detrusor was then closed on top of the distal ureter using interrupted Vicryl 2/0 sutures.

When necessary, a psoas hitch was performed to ensure a tension‐free anastomosis by mobilizing and securing the bladder to the psoas muscle with a V‐Lock 3/0 to spread the tension gradually. Care was taken to place the sutures longitudinally to the psoas muscle and not too deeply to minimize the risk of nerve injury.

Side‐to‐side reimplantation was another option for distal ureteral strictures. In this technique, both the bladder wall and the ureter were incised on their lateral aspects to avoid transecting the ureter. The ureter was then directly anastomosed to the bladder without creating an antireflux mechanism. This technique was elected in comorbid patients or difficult surgical environments to decrease the operative time and try to optimize the ureteral vascularization by avoiding transection.

#### 2.2.2 Open reimplantation

For open reimplantation, a midline incision with a transperitoneal approach or a Gibson incision with an extraperitoneal approach was used based on the surgeon's preference. The ureter was identified and carefully dissected. The ureter was always transected and reimplanted without bladder mobilization if the stricture was distal enough to achieve a tension‐free anastomosis (ureteroneocystostomy).

When necessary, a psoas hitch was performed to ensure a tension‐free anastomosis by mobilizing and securing the bladder to the psoas muscle with a permanent suture (Ethibond 2/0).

The decision to perform a refluxing vs non‐refluxing reimplantation was left to the surgeon's discretion. When an antireflux reimplant was elected a Lich‐Gregoir or a Leadbetter–Politano antireflux mechanism was created depending on the surgeon's preference.

A ureteral stent was placed before the anastomosis was completed for both open and robotic reimplantation and was removed at 4 to 8 weeks.

All patients had imaging at 3 months from the surgery which was either a CT scan or an ultrasound. In case of hydronephrosis, further investigations were performed if the patient had symptoms (pain, infection, decline in renal function …).

Emergency reimplantations performed intraoperatively upon immediate request for traumatic surgical ureteral injuries were excluded, as these urgent procedures lacked prior ureteral preparation and optimal surgical conditions. Their inclusion could have introduced confounding bias.

### 2.3 Outcomes of interest

The primary outcome was the stricture recurrence rate. Given the lack of a standardized definition in the literature, a composite criterion was established, including any of the following events: need for urinary drainage, reoperation or symptomatic upper urinary tract dilatation observed at the last follow‐up. A stricture was considered recurrent if any of these conditions were met. The secondary outcomes of interest were the occurrence of early postoperative complications, defined as any complication occurring within 30 days following surgery graded according to the Clavien–Dindo classification, and all the main perioperative outcomes (operative time, length of hospital stay, …).^9^ The following long‐term complications were also collected and analysed: recurrent urinary tract infections, symptomatic reflux, de novo lower urinary tract symptoms and unexplained flank pain (i.e. flank pain in the absence of stricture recurrence or symptomatic reflux).

### 2.4 Statistical analysis

Means and standard deviations were reported for continuous variables, medians and ranges for categorical variables and proportions for nominal variables. Comparisons between groups were performed using the χ^2^ test or Fisher's exact test for discrete variables and the Mann–Whitney test for continuous variables as appropriate. Univariate logistic regression analysis was conducted to seek predictors of stricture recurrence. Statistical analyses were performed using JMP Pro v.18.0.1 software (SAS Institute Inc., Cary, NC, USA). All tests were two‐sided with *p* < 0.05 as a threshold to define statistical significance.

## 3 RESULTS

### 3.1 Patients' characteristics

**FIGURE 1:**
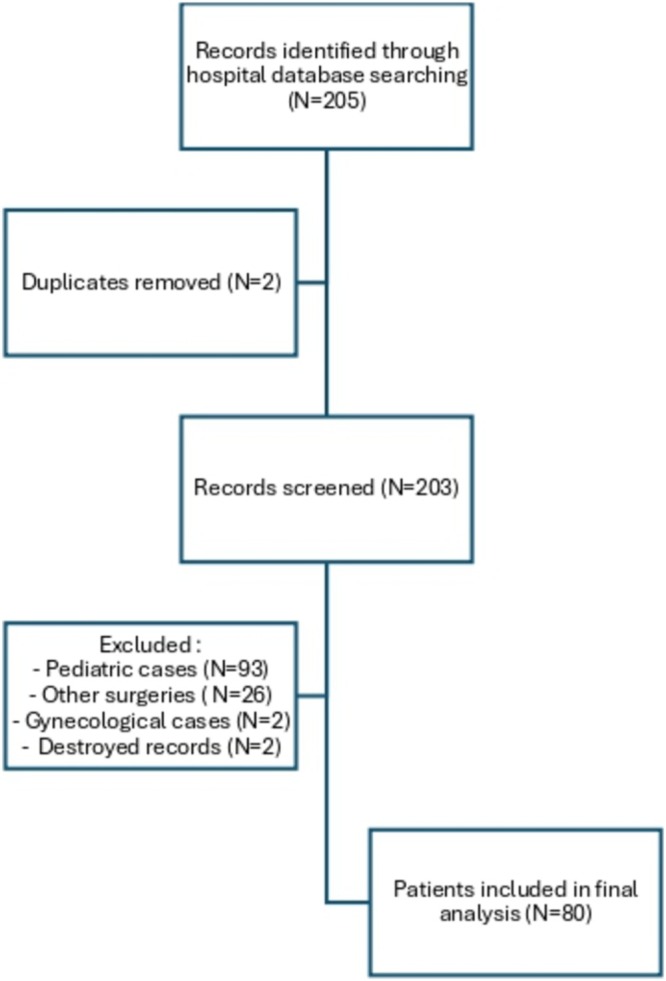
Flowchart.

An initial screening identified 205 patients. Following the eligibility assessment, 80 patients were included in the final analysis: 45 in the open surgery group and 35 in the robotic group (Figure 1). In both groups, procedures were performed by junior surgeons under the supervision of one of three senior reconstructive urologists; a total of 15 and 14 surgeons were involved in the open and robot‐assisted groups, respectively. The patients' characteristics are summarized in Table 1. The open and robotic groups were comparable for most variables. There was a higher proportion of women in the robotic group (88.6% vs. 62.2%;p = 0.01), and the number of patients having undergone prior endoscopic treatments was higher in the robotic group as well (28.9% vs. 8.9%; p = 0.04). No bilateral ureteral strictures were included in the present series.

**TABLE 1:** Patients' preoperative characteristics.

	Open group (N = 45)	Robotic group (N = 35)	p‐value
**Gender, n (%)** Female Male	28 (62.2) 17 (37.8)	31 (88.6) 4 (11.4)	**p = 0.01**
**Median age at surgery, years (IQR)**	49 (40–63)	49 (37–60)	p = 0.77
**Median BMI, kg/m** ^**2**^ **(IQR)**	25.4 (+/−7)	24.7 (+/−5)	p = 0.41
**Previous pelvic surgery, n(%)**	37 (84.1)	29 (82.9)	p = 0.97
**Stricture location, n (%)** Iliac ureter Pelvic ureter	5 (11.1) 40 (88.9)	1 (2.9) 34 (97.1)	p = 0.16
**Previous radiotherapy, n(%)**	6 (13.3)	4 (11.4)	p = 0.80
**Ureteral rest > 4 weeks, n(%)**	36 (80)	28 (80)	p = 0.99
**Prior endoscopic treatment of the stricture, n (%)**	4 (8.9)	10 (28.9)	**p = 0.04**
**Prior attempt of surgical repair, n (%)**	3 (9.4)	3 (8.6)	p = 0.91

### 3.2 Surgical techniques, stricture outcomes and long‐term complications

The surgical techniques and stricture outcomes are presented in Table 2. There were more psoas hitches in the open group (75.6% vs. 48.6%; p = 0.02) and more side‐to‐side reimplantation in the robotic group (14.3% vs. 2.2%; p = 0.02). The vast majority of reimplantation was non‐refluxing (88.9% vs. 74.3%; p = 0.08). Intravenous indocyanine green was used in 40% of patients in the robotic group vs. 0 in the open group (p < 0.0001). After a median follow‐up duration of 16.5 months for the open group and 10 months for the robotic group, the stricture recurrence rate was similar in both groups (6.8% vs. 8.6%; p = 0.99). The six strictures recurrence (7.5% of the whole cohort) occurred within the first 4 months (median time to recurrence 2 months). In terms of long‐term complications, there were similar rates of symptomatic reflux (4.4% vs. 5.7%; p = 0.99) and flank pain (8.9% vs. 8.6%; p = 0.99) between both groups. There were more recurrent urinary tract infections in the open group (17.8% vs. 8.6%) and more de novo lower urinary tract symptoms in the robotic group (11.4% vs. 2.2%), but these differences were not statistically significant (p = 0.33 and p = 0.16, respectively). There were more symptomatic reflux when a refluxing reimplantation was performed (14.3% vs. 3.1%) but this difference was not statistically significant (p = 0.15) The long‐term complications rates for the whole cohort were 7.5% for stricture recurrence, 5% for symptomatic reflux, 8.8% for flank pain, 6.3% for de novo lower urinary tract symptoms (LUTS), 13.8% for recurrent urinary tract infections. Overall, 30 patients (38%) had at least one long‐term complication (35.6% vs 40%; p = 0.82).

**TABLE 2:** Surgical technique, stricture outcomes and long‐term complications.

	Open group (N = 45)	Robotic group (N = 35)	p‐value
**Technique, n (%)** Ureteroneocystostomy Psoas hitch Side‐to‐side	10 (22.2) 34 (75.6) 1 (2.2)	13 (37.1) 17 (48.6) 5 (14.3)	**0.02**
**Type of reimplantation, n (%)** Refluxing Non‐refluxing	5 (11.1) 40 (88.9)	9 (25.7) 26 (74.3)	0.08
**Intravenous indocyanine green, n (%)**	0 (0)	14 (40)	**<0.0001**
**Intraoperative ureteroscopy, n (%)**	0 (0)	4 (11.4)	0.03
**Symptomatic reflux, n (%)**	2 (4.4)	2 (5.7)	0.99
**Flank pain, n (%)**	4 (8.9)	3 (8.6)	0.99
**De novo lower urinary tract symptoms, n(%)**	1 (2.2)	4 (11.4)	0.16
**Recurrent urinary tract infections, n(%)**	8 (17.8)	3 (8.6)	0.33
**Stricture‐free patients at last follow up** **°** **, n(%)**	41 (93.2)	32 (91.4)	0.99
**At least one long‐term complication (stricture, reflux, UTI, pain, LUTS), n(%)**	16 (35.6)	14 (40)	0.82

° One patient in the open group was excluded because of death at 7 months postoperatively.

### 3.3 Perioperative outcomes

The perioperative outcomes are detailed in Table 3. The mean estimated blood loss was lower in the robotic group (111.2 vs. 242.9 ml; p = 0.03) and so was the median length of hospital stay (3 vs 11 days; p < 0.0001). The overall complications' rates were comparable in both groups (40% vs. 53.3%; p = 0.24). The proportions of major complications (Clavien grade 3 or higher) did not differ significantly between the two groups (5.7% vs. 8.9%; p = 0.24), but there was one death in the open group. Early reoperations (Clavien‐Dindo grade 3b, <30 days) occurred in two patients in each group: in the open group, one required nephrostomy for obstructive pyelonephritis and one underwent early open revision for anastomotic leakage; in the robot‐assisted group, one required nephrostomy for obstructive pyelonephritis and one underwent JJ stent removal under general anaesthesia due to severe pain. Analgesic requirements were not systematically recorded due to their self‐reported nature. None of the patients were discharged with opioids, and postoperative pain control was therefore indirectly reflected by hospital stay duration. Five patients required readmission for pain management.

**TABLE 3:** Perioperative outcomes.

	Open group (N = 45)	Robotic group (N = 35)	p‐value
**Mean operative time (minutes, +/− SD)**	186.2 (+/−83.5)	197.9 (+/−81.3)	0.52
**Mean estimated blood loss (cc, +/− SD)**	242.9 (+/−205.6)	111.2 (+/− 162.7)	**0.03**
**Median hospital stay duration (days, IQR)**	11 (8–14)	3 (2–6)	**p < 0.0001**
**Ureteral stent left postoperatively, n(%)**	44 (97.8)	34 (97.1)	0.99
**Median foley catheter drainage duration, days (IQR)**	9 (7–12)	8 (6–12)	0.54
**Complications, n(%)** Clavien grade 1 2 3b 4 5	24 (53.3) 8 12 2 1 1	14 (40) 5 7 2 0 0	0.24

### 3.4 Predictive factors of stricture recurrence

As seen in Table S4, in univariate logistic regression analysis, the robotic approach was not associated with the risk of stricture recurrence (OR = 1.28; p = 0.77). The only variable significantly associated with the risk of stricture recurrence was radiotherapy (OR = 11.2; p = 0.01). The recurrence rate was 30% in radiated patients vs. 4.3% in non‐radiated patients (p = 0.01);

## 4 DISCUSSION

Ureteral reimplantation has been considered the gold‐standard surgical repair for ureteral stricture for a long time.^10^, ^11^, ^12^ The robotic approach has emerged over the past decade as an alternative to open or laparoscopic ureteral reimplantation but the head‐to‐head comparison in the literature remains relatively limited.^7^, ^8^, ^13^ In the present series, one of the largest published to date, we observed that the open and robotic approaches were comparable in terms of stricture recurrence rates but with some advantages for the robotic approach in terms of perioperative morbidity.

These findings underscore the possible role and potential of robot‐assisted laparoscopy compared to open surgery for ureteral reconstruction. Several studies and reviews have already highlighted the benefits of robotic surgery, particularly in reducing hospital stay and blood loss.^14^, ^15^, ^16^ The robotic platform offers enhanced visualization—further improved by adjuncts such as indocyanine green imaging and intraoperative ureteroscopy—as well as superior dexterity. This combination may enable more precise dissection and better preservation of ureteral vascularization. It may also shorten the learning curve by all the educational opportunities it provides, with a good vision of the procedure for the trainee, the dual console, simulators, … Although the robotic approach appeared to improve the perioperative morbidity, one may argue that this could also reflect the impact of the overall learning curve from the centre. The surgical team may have improved its understanding of the anatomy and surgical techniques over the year which may have benefited the robotic group as in any study comparing a historical to a contemporary cohort. It is important to stress that, although the length of stay and estimated blood loss matter, they remain relatively “weak” outcomes. Considering the absence of significant differences in stricture recurrence and major complications, our study certainly did not prove a real superiority of robotic over open ureteral reimplantation.

In this series, 38% of patients experienced long‐term complications such as lower urinary tract symptoms (LUTS) or symptomatic reflux. This underscores the overall morbidity associated with ureteral reimplantation, which alters the native bladder anatomy and raises the ongoing debate between refluxing versus non‐refluxing reimplantation for distal ureteral stenosis. A promising and increasingly attractive alternative emerging in the field of robotic ureteral reconstruction is the use of buccal mucosa grafts (BMG). A comparative analysis between reimplantation and BMG for distal ureteral strictures could be valuable, as BMG may offer a lower incidence of long‐term complications.^17^

This issue of unstandardized follow‐up is a possible underestimation of the stricture recurrence rate and symptomatic reflux recurrence rate. However, all recurrence occurring within the first 4 months suggests that long‐term follow‐up might not be needed. Postoperative imaging (CT or renal scan) was not systematically performed in asymptomatic patients, as isolated hydronephrosis was not considered indicative of recurrence.

One of the key takeaways from this study is that a history of radiation therapy emerged as a significant risk factor for recurrence. Patients with prior radiation should therefore undergo thorough preoperative evaluation and be counselled regarding the increased risk of treatment failure.

Another important consideration in ureteral reconstruction is the role of ureteral rest. Its efficacy in reducing the risk of recurrence in ureteral strictures has been demonstrated in the literature. In the present study, patients in both groups underwent prolonged ureteral rest—exceeding four weeks—with no significant difference in its prevalence between the groups. Consequently, ureteral rest was not included in the multivariable analysis, as it was unlikely to have introduced bias or significantly influenced the outcomes.

This study has several limitations that should be acknowledged. First, it has all the biases inherent to its retrospective single‐centre design. The inclusion of all open procedures in the open group—including those performed after the introduction of the surgical robot at our centre—may introduce confounding bias. In such cases, open surgery may have been preferentially selected for patients with significant comorbidities or anticipated surgical complexity that contraindicated robotic intervention. Being conducted at a teaching hospital, the study involved many different surgeons with various levels of expertise, which could not be accounted for and may represent a significant confounder. The decision‐making process for selecting between open and robot‐assisted approaches was not standardized, particularly after the introduction of robotic surgery, which represents a limitation of the study. Cost analysis was not performed in this study; however, future work comparing the expenses related to robotic platform use with those associated with longer hospitalization in open surgery would be of great interest.

Although long‐term complications were identified as an important finding, their evaluation herein is prone to several biases as no standardized follow‐up protocol nor standardized questionnaire was used. Finally, although this series is one of the largest comparisons available to date, its sample size remains relatively limited, especially considering the heterogeneity of patients' profiles and surgical techniques used.

## 5 CONCLUSION

The robotic approach appears to be non‐inferior to the open approach in terms of stricture recurrence while being associated with a shorter length of hospital stay and lower estimated blood loss. This study confirms the higher risk of failure in radiated patients. More importantly, the present series raises questions regarding the long‐term consequences of ureteral reimplantation with significant rates of flank pain, symptomatic reflux, de novo LUTS and recurrent urinary tract infections regardless of the surgical approach. These long‐term complications have been poorly examined so far in the literature and warrant further investigations. This may reconsider the role of ureteral reimplantation as the gold‐standard surgical repair for lower ureter stricture while buccal mucosa graft ureteroplasty concomitantly emerges as a promising alternative.

## AUTHOR CONTRIBUTIONS

*Acquisition of data*: Alice Bourillon. *Analysis and interpretation of data*: Alice Bourillon, Benoit Peyronnet. *Drafting of the manuscript*: Alice Bourillon, Benoit Peyronnet, Karim Bensalah, Lee C. Zhao. *Critical revision of the manuscript for important intellectual content*: Alice Bourillon, Lucas Freton, Gregory Verhoest, Juliette Hascoet, Claire Richard, Camille Haudebert, Romain Mathieu, Lee C. Zhao, Karim Bensalah, Benoit Peyronnet. *Statistical analysis*: Alice Bourillon, Benoit Peyronnet. *Administrative, technical, or material support*: Alice Bourillon, Benoit Peyronnet. *Supervision*: Benoit Peyronnet, Karim Bensalah, Lee C. Zhao.

## CONFLICT OF INTEREST STATEMENT

The authors have no conflict of interest to disclose.

## Supporting information


**Table S4:** Predictive factors of stricture recurrence in univariate regression analysis.
