# Supplementary material for: Robotic vs. open ureteral reimplantation: A retrospective comparative single‐centre series
Source: BJUI Compass. 2025 Nov 16;6(11):e70110. doi: 10.1002/bco2.70110 (PMC12620033; doi:10.1002/bco2.70110)
Supplement: Supplementary file 1 — Table S4: Predictive factors of stricture recurrence in univariate regression analysis. [file BCO2-6-e70110-s001.pdf]

Variable	Odds Ratio	CI95%	p-value
**Ureteral rest**	1.29	0.19-25.74	0.82
**Gender (female vs male)**	1.76	0.26-34.83	0.60
**Previous failed surgical repair**	2.24	0.11-18.15	0.53
**Previous failed endoscopic treatment**	2.5	0.32-14.41	0.32
**Robot vs open**	1.28	0.22-7.32	0.77
**Body Mass Index**	1.02	0.85-1.19	0.86
**Age**	1.04	0.97-1.10	0.24
**History of radiotherapy**	11.2	1.75-73.74	**0.01**
**Type of reimplantation**<br>**Non-refluxing**<br>**refluxing**	 1 (Ref)<br>0.91	 <br>0.05-6.28	 <br>0.93
**Surgical technique**<br>**Ureteroneocystostomy**<br>**Psoas Hitch**<br>**Side to side**	 1 (Ref)<br>2.44<br>0.20	 <br>0.36-48.30<br>0.02-23.44	 <br>0.99<br>0.99

Table 4: Predictive factors of stricture recurrence in univariate regression analysis
